# Paracoccidioidomycosis presenting as a cecal lesion mimicking cholangiocarcinoma: a case report of unusual intestinal manifestation

**DOI:** 10.1590/S1678-9946202466063

**Published:** 2024-11-11

**Authors:** Eduarda Raunheitti Giesteira, Giovanna França Santore, Julia de Abreu Teixeira, Ezequias Batista Martins, Bianca Balzano de la Fuente Villar, Billy McBenedict, Karla Regina Oliveira de Moura Ronchini, Natalia Chilinque Zambão da Silva, Nilo Fernandes Leça, Patrícia Yvonne Maciel Pinheiro, Remberto Maurício de La Cruz Vargas Vilte, Thais de Oliveira Vieira, Laura da Cunha Ferreira

**Affiliations:** 1 Universidade Federal Fluminense, Faculdade de Medicina, Niterói, Rio de Janeiro, Brazil; 2 Hospital Universitário Antônio Pedro, Serviço de Infectologia, Niterói, Rio de Janeiro, Brazil; 3 Hospital Universitário Antônio Pedro, Serviço de Radiologia, Niterói, Rio de Janeiro, Brazil

**Keywords:** Paracoccidioidomycosis, *Paracoccidioides* spp, Appendicitis, Acute abdomen

## Abstract

Paracoccidioidomycosis, the most important systemic mycosis in Latin America, is closely linked to rural activities. In Brazil, it is an endemic disease, with an estimated 4,000 to 6,000 annual cases, accounting for over 80% of the global diagnoses. We present an intriguing case of this disease with an intestinal manifestation in a 71-year-old woman. The involvement of the cecal appendix led to a complication of cholangitis that mimicked cholangiocarcinoma.

## INTRODUCTION

Paracoccidioidomycosis (PCM) primarily affects tropical regions in Brazil, Argentina, Colombia, Ecuador, and Venezuela^1^. Traditionally, the disease is more prevalent among adult men engaged in agricultural activities. However, there is a growing incidence in urban areas, with an increasing number of cases among women^2^. The causative agent is a thermodimorphic fungus of the genus *Paracoccidioides* spp^3^.

The fungus typically enters the human body through inhalation during childhood as infectious propagules (conidia). In the lungs, it forms a primary complex, which can potentially disseminate to other organs via lymphatic and hematogenous systems. Over time, the infection may progress, leading to chronic forms of the disease in adults. Although uncommon (3% to 5% of cases)^4^, acute and subacute forms can occur in childhood and adolescence, referred to as the juvenile type.

Intestinal PCM predominantly affects the jejunum, ileum, and proximal portions of the large intestine. Generally, it causes a chronic inflammatory process, simulating Crohn's disease^5^. We report a notable case of this disease with intestinal presentation in a 71-year-old woman without lung involvement. The disease affected her cecal appendix and led to a complication of cholangitis that mimicked cholangiocarcinoma.

## CASE REPORT

**Figure 1:**
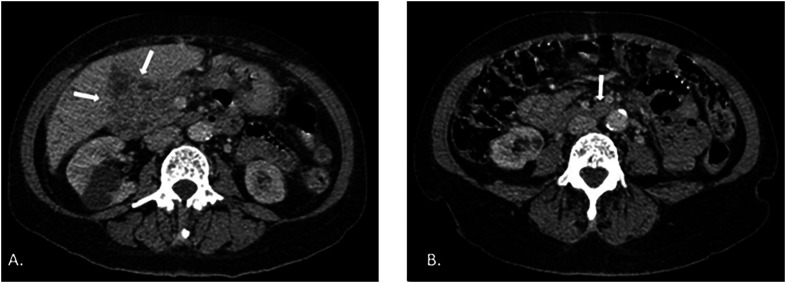
Abdominal CT performed before treatment: (A) peribiliary infiltrative tissue associated with perihepatic lymph node enlargement; (b) Prominent retroperitoneal lymph nodes.

In June 2023, a healthy 71-year-old hypertensive Brazilian woman presented with acute icteric syndrome, which manifested as abdominal pain, diarrhea, vomiting, and severe weight loss (20 kg in three months). Outpatient clinical investigation was unable to reach a diagnosis in the first months of the disease and the patient was treated only with symptomatic medications. Then, after five months, her abdominal pain worsened, leading to a confirmed diagnosis of appendicitis. She underwent an emergency appendectomy. Histopathological examination of the cecal appendix revealed acute appendicitis with a lymphohistiocytic infiltrate with multinucleated giant cells showing fungal spores with peripheral budding in hematoxylin and eosin staining. A tuberculosis test of the sample returned negative results. Preoperative abdominal computed tomography (CT) suggested a possible diagnosis of cholangiocarcinoma (Figure 1).

In February 2024, Magnetic Resonance Cholangiography (MRC) revealed nodular thickening in the gallbladder wall. Multiple adenomegaly were observed in the hepatic hilum and retroperitoneum. Additionally, nodules were detected in the left adrenal gland, suggestive of metastatic lesions. The examination findings strongly suggested cholangiocarcinoma with potential metastases. However, the changes observed in the gallbladder, adrenal gland, and abdominal lymph nodes were hypothesized to be fungal abscesses, evidenced by the previous confirmation of fungi in the removed cecal appendix. The patient had no previous history of pulmonary or mucocutaneous disease that would suggest a diagnosis of PCM. Radiological examination of the lungs was normal.

On March 5, 2024, the patient was hospitalized with jaundice, with potential diagnoses of PCM and bile duct cancer. Upon admission, she presented with jaundice (+/4+) and lymphadenopathy in the cervical, submental, inguinal, and axillary regions. The swollen nodes exhibited no neoplastic pattern. Based on the evidence of active PCM, she was treated with amphotericin B deoxycholate (50 mg/day) for nine days, followed by liposomal amphotericin B (50 mg/day) for 21 days, totaling 30 days of antifungal treatment.

During hospitalization, a blood culture test showed the growth of *Escherichia coli*. The patient was prescribed ceftriaxone and metronidazole for eight days. Following episodes of bleeding (melena and hematemesis), an endoscopic examination revealed gastric ulcers, which required clipping of the ulcers. On March 15, 2024, a liver lesion biopsy was performed using interventional radiology, and the histopathological examination results were negative for cancer.

**Figure 2:**
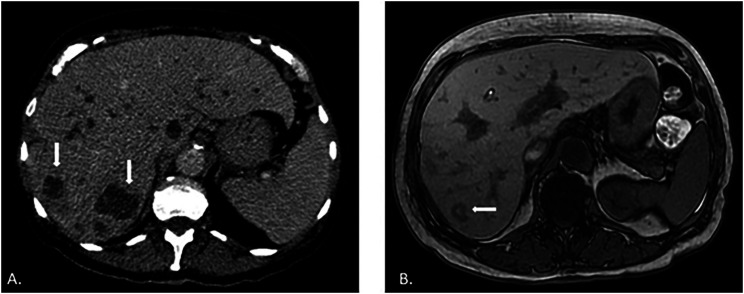
(A) Abdominal CT performed before treatment, showing multiple lobulated expansive formations in the right lobe of the liver, the largest measuring 3.4 cm; (B) Abdominal MRI performed after treatment, showing a nodular image with hyposignal on T1, located in segment VII of the liver, measuring 1.8 cm (smaller compared to the previous tomographic image).

After 30 days of treatment with amphotericin B (50 mg/day), the patient showed clinical and radiological improvement (Figure 2). A follow-up MRC revealed significant improvement in the biliary lesions, effectively ruling out the possibility of cancer. On April 9, 2024, the patient was discharged from the hospital with a treatment plan to continue oral itraconazole (200 mg/day) for one year.

Informed consent was obtained from all the subjects involved in the study. The research ethics committee of the Hospital Universitario Antonio Pedro reviewed and approved the study and assigned the approval Nº CAAE: 79871924.1.0000.5243.

## DISCUSSION

PCM predominantly impacts the lungs and skin. Extrapulmonary presentation is rare, occurring in less than 1% of cases, and is associated with significant mortality^6^. Atypical cases of paracoccidioidomycosis have been described, with involvement of the adrenal glands, prostate, and central nervous system. However, all cases showed concomitant pulmonary involvement^7,8^. In this case, the patient had no prior pulmonary or cutaneous manifestations, which initially made the diagnosis of PCM seem unlikely. It was only via histopathological examination of the removed cecal appendix that the diagnosis of PCM was considered.

An important study demonstrated that culture is positive in only 30% of cases of fungal diseases diagnosed by histopathology^9^. Additionally, research conducted in two Spanish hospitals found that only 56% of positive fungal diagnoses by histopathology were confirmed by microscopic examinations (direct examination and culture)^10^. In this case, the histopathological examination of the cecal appendix led to the diagnosis of PCM, enabling adequate treatment for the patient, despite negative fungal culture tests.

The intestinal form is rare, with lymph nodes, joints, bones, mediastinum, and meninges being the most frequently affected organs. In the abdomen, besides the intestine, it can cause lymphadenopathy, hepatomegaly, splenomegaly, and in some cases, even affect the gallbladder^11^. Clinically, the abdominal form manifests as localized pain, diarrhea, secondary appendicitis, colitis, and colon ulcers^12^. Although all segments of the digestive tract can be affected, lesions are more commonly found in regions rich in lymphoid tissue, such as the terminal ileum and appendix^13^. In this case, appendicitis occurred due to the lymphoid involvement of the organ by the fungus. The prompt diagnosis of appendicitis was crucial in saving the patient's life, and the examination of the removed appendix led to the diagnosis of PCM.

Reports of intestinal PCM causing acute abdomen are rarely described in the literature. In Brazil, a case of a juvenile form was reported, which progressed to necrosis of mesenteric lymph nodes, constituting a surgical emergency^14^. Giron *et al*.^15^ documented a case of PCM in a 73-year-old man with severe involvement of the jejunum, leading to necrosis and requiring partial intestinal resection. In patients with intestinal PCM, computed tomography may show marked ileocecal wall thickening, sometimes appearing as a mass, and is often associated with a conglomerate group of enlarged lymph nodes^12,16^. This case report underscores the importance of including PCM in the differential diagnosis of acute abdomen in endemic regions, highlighting the need for prompt and effective treatment.

## CONCLUSION

The reported case highlights an unusual and serious presentation of intra-abdominal involvement by *Paracoccidioides* spp. in a female patient and reinforces the importance of compulsory notification of mycoses. Moreover, this case emphasizes the need to consider PCM as a potential differential diagnosis for acute abdomen in endemic regions, such as Southeastern Brazil.
